# Small molecule inhibits T-cell acute lymphoblastic leukaemia oncogenic interaction through conformational modulation of LMO2

**DOI:** 10.18632/oncotarget.27580

**Published:** 2020-05-12

**Authors:** Leanne Milton-Harris, Mark Jeeves, Sarah A. Walker, Simon E. Ward, Erika J. Mancini

**Affiliations:** ^1^School of Life Sciences, Biochemistry Department, University of Sussex, Falmer, Brighton, BN1 9QG, United Kingdom; ^2^Institute of Cancer and Genomic Sciences, College of Medical and Dental Sciences, University of Birmingham, Edgbaston, Birmingham, B15 2TT, United Kingdom; ^3^Sussex Drug Discovery Centre, University of Sussex, Brighton, BN1 9QJ, United Kingdom; ^4^Medicines Discovery Institute, Cardiff University, Park Place, Cardiff, CF10 3AT, United Kingdom

**Keywords:** protein-protein interaction, leukaemia, T-ALL, drug discovery, LMO2

## Abstract

Ectopic expression in T-cell precursors of LIM only protein 2 (LMO2), a key factor in hematopoietic development, has been linked to the onset of T-cell acute lymphoblastic leukaemia (T-ALL). In the T-ALL context, LMO2 drives oncogenic progression through binding to erythroid-specific transcription factor SCL/TAL1 and sequestration of E-protein transcription factors, normally required for T-cell differentiation. A key requirement for the formation of this oncogenic protein-protein interaction (PPI) is the conformational flexibility of LMO2. Here we identify a small molecule inhibitor of the SCL-LMO2 PPI, which hinders the interaction *in vitro* through direct binding to LMO2. Biophysical analysis demonstrates that this inhibitor acts through a mechanism of conformational modulation of LMO2. Importantly, this work has led to the identification of a small molecule inhibitor of the SCL-LMO2 PPI, which can provide a starting point for the development of new agents for the treatment of T-ALL. These results suggest that similar approaches, based on the modulation of protein conformation by small molecules, might be used for therapeutic targeting of other oncogenic PPIs.

## INTRODUCTION

Stem cell leukaemia (SCL, also known as TAL-1) is a basic helix-loop-helix transcription factor with essential, non-redundant roles in haematopoietic development and the terminal maturation of erythroid cell lineages [1, 2]. Central to SCL function is the ability to bind the haematopoietic transcription co-factor LIM-only protein 2 (LMO2) [3]. LMO2 has multiple essential roles in erythroid differentiation, angiogenesis and CNS development [4–10]. Aberrant LMO2 expression due to chromosomal translocations or interstitial deletions [11–13] has been observed in multiple haematological malignancies, most notably T-cell acute lymphoblastic leukaemia (T-ALL) [4, 14]. Ectopic expression of SCL through chromosomal translocations or microdeletion or transactivation through abnormal expression of proteins within the haematopoietic regulatory network [15] is observed in ~60% of T-ALL cases of which ~40% also display abnormal LMO2 expression [10, 11]. The two proteins have been well characterised as acting synergistically to promote a phenotype of aberrant self-renewal in T-cell precursors through formation of a multiprotein complex with essential roles in both normal and malignant haematopoiesis [3, 16, 17], driving a mechanism of E2A protein sequestration [10, 11, 16]. Additionally, dysregulation of LMO2 alone through somatic mutation [18] or resulting from retroviral activation mutagenesis following treatment for X-SCID [19] is recognised as an oncogenic promoter in T-ALL.

Studies of the pre-leukemic phenotype in mouse models suggest that both SCL and LMO2 overexpression causes T-ALL by inducing aberrant self-renewal of committed T-cells in the thymus, resulting in a cellular pool which can over time acquire additional gain-of-function mutations of genes involved in signalling pathways regulating T-cell development, such as NOTCH1, interleukin-7, KIT and FLT3 [20]. Relapse is associated with clonal evolution from a population of pre-leukemic stem cells that acquire the whole set of malignant mutations leading to a full-blown T-ALL. Numerous studies have suggested that SCL and LMO2 function cooperatively to drive this phenotype of aberrant self-renewal in T-cell precursors through a mechanism of functional sequestration of E2A-protein homodimers which are essential for normal T-cell differentiation [10, 11, 16, 21–26]. As the binding affinity of LMO2 for SCL/E2A-containing complexes is very high (K_a_ = 1.8 × 10^8^ M^-1^), the sequestration of E2A proteins is strongly favoured in cells that express both SCL and LMO2 [27]. Current therapeutic options for the treatment of T-ALL are limited to an extended and aggressive program of cytotoxic chemotherapies and radiotherapies. While effective, these treatments are associated with significant, long-term adverse effects, particularly in young children. Additionally, patients presenting with relapsed T-ALL have a poor prognosis with current treatments. Targeting the critical SCL-LMO2 protein-protein interaction (PPI) provides an alternative approach to developing novel therapeutics for these patients.

We have previously solved the atomic structure of the SCL_bHLH_/E47_bHLH_ – LMO2:LDB1^LID^ quaternary complex (hereafter referred to as the SCL-LMO2 complex) using X-ray crystallography [16] and mapped the binding interface by mutagenesis. This revealed that only a small number of amino acids contribute to the binding interface and that flexibility around hinge amino acid F88 is crucial for this PPI *in vitro*, *in vivo* and *ex vivo* [16, 28, 29]. Within this structural framework, we have used a combination of biophysical and biochemical techniques to screen for small molecules with the goal of developing compounds which can specifically inhibit the SCL LMO2 PPI. Using a homogeneous time-resolved fluorescence (HTRF) assay we have identified a dose-responsive hit compound (3K7), which inhibits the SCL-LMO2 PPI *in vitro*. We have used Saturation Transfer Difference Nuclear Magnetic Resonance (STD-NMR) and Microscale Thermophoresis (MST) to demonstrate that 3K7 binds LMO2 with 1.2 μM affinity. Using Small Angle X-ray Scattering (SAXS) we demonstrate that 3K7 acts through a mechanism of structural modulation, locking LMO2 in a conformation that inhibits its interactions with SCL. Our work suggests that developing small molecules interfering with the conformational flexibility of LMO2 is a realistic and novel approach for the therapeutic targeting of T-ALL.

## RESULTS

### HTRF screen identifies dose-dependent inhibitors of SCL-LMO2 PPI

**Figure 1:**
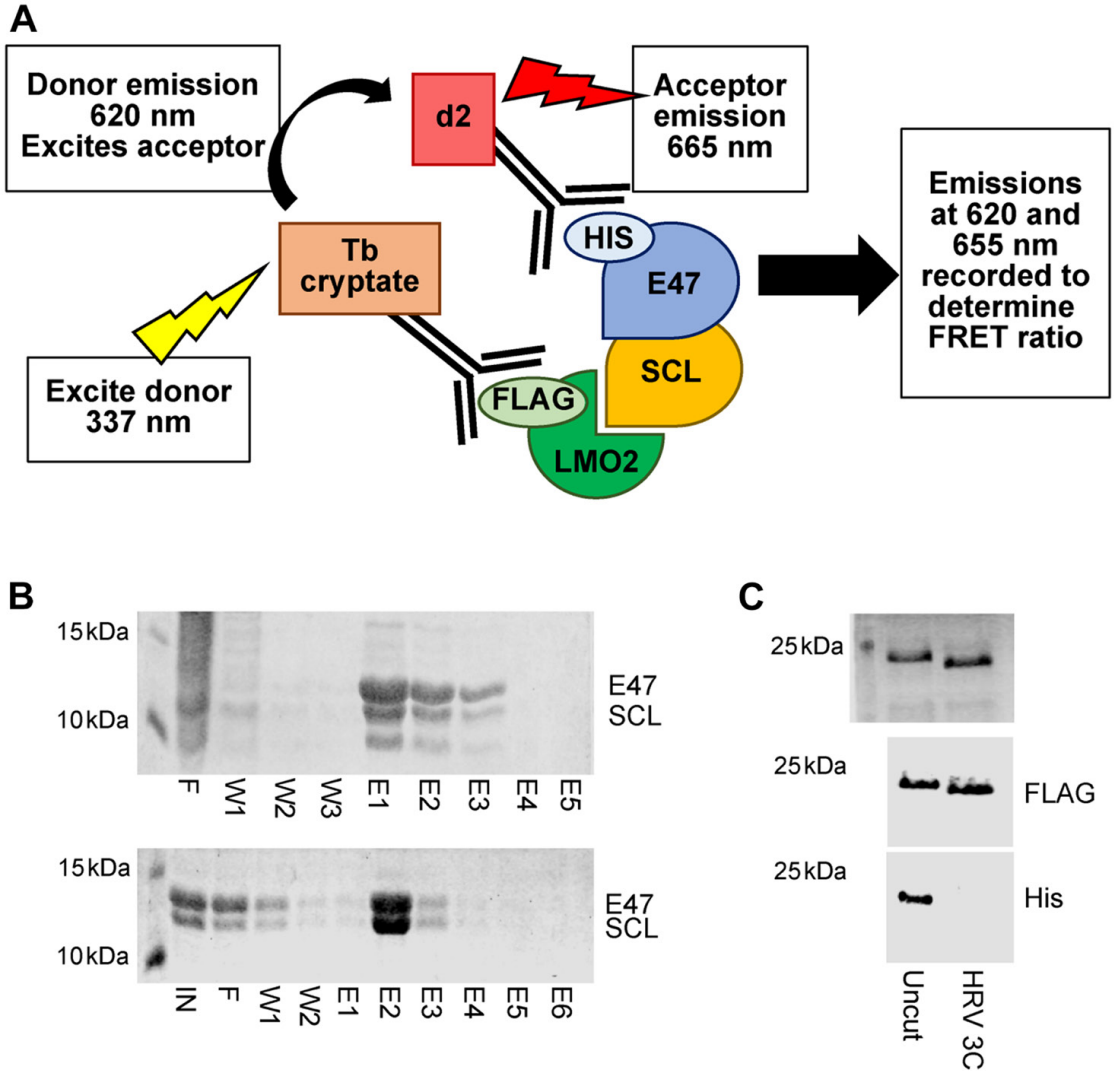
Primary HTRF assay set up. (**A**) Schematic illustrating the principle of the HTRF assay. Formation of the quaternary protein complex SCL/E47-LMO2:LDB1^LID^ brings affinity antibody conjugated fluorophores into proximity. Subsequent excitation of the donor fluorophore results in energy transfer and excitation of the acceptor fluorophore. Emissions from both fluorophores can be detected and quantified to determine the FRET ratio. Disruption of complex formation by small molecule binding results in a quantifiable reduction in FRET signals. (**B**) SDS-PAGE analysis of SCL-E47 heterodimer purification. Top panel: Fractions from His purification showing mixed homo- and hetero-dimers. Bottom panel: Fractions from strep purification showing isolated heterodimers. IN = input, F = flow through, W = wash, E = elution. (**C**) SDS-PAGE and Coomassie staining (top) or western blotting with anti-FLAG (middle) or anti-His (bottom) antibodies show complete removal of an N-terminal His tag from LMO2:LDB1^LID^-FLAG following incubation with HRV 3C protease (uncut vs. 3C treated lanes).

To identify initial inhibitors of the SCL-LMO2 interaction *in vitro*, a homogeneous time-resolved fluorescence (HTRF) assay was established, using fluorophores conjugated to anti-His and anti-FLAG affinity antibodies to observe protein interactions (Figure 1A) [30]. Recognition of the affinity tags on the purified proteins by the antibodies acts to effectively fluorescently label each protein without compromising the interaction interface. Using anti-FLAG terbium cryptate as a donor fluorophore and anti-His-d2 as the acceptor fluorophore allows for detection of red-shifted fluorescence signals at a time delay, reducing background signals from compound autofluorescence and improving signal to noise ratios for the assay.

SCL forms obligate heterodimers with its binding partner E47 through the bHLH domains of each protein. Co-expression of the SCL_bHLH_ and E47_bHLH_ domains results in a mixed population of SCL-E47 heterodimers and E47 homodimers, which are indistinguishable by SEC. Cloning of an N-terminal Strep-II tag on SCL_bHLH_ and purification with Strep-Tactin allowed isolation of the SCL-E47 heterodimer species (Figure 1B) which was subsequently labelled with anti-His affinity antibody conjugated fluorophores. The LMO2:LDB1^LID^ fusion protein previously used for structure determination by X-ray crystallography [16, 28, 31, 32] was cloned into a vector with a cleavable N-terminal 6xHis tag and a C-terminal FLAG tag. Following initial IMAC purification and cleavage of the His tag, the protein was isolated by size exclusion chromatography (SEC) prior to specific labelling of the protein with anti-FLAG antibody conjugated fluorophores (Figure 1C).

Upon formation of the core quaternary complex (LMO2:LDB1^LID^-SCL_bHLH_/E47_bHLH_), the two fluorophores are brought into close proximity. Subsequent excitation of the terbium cryptate donor then results in Förster Resonance Energy Transfer (FRET) leading to detectable emissions from both fluorophores. Disruption of the complex through inhibition of the protein-protein interaction thus leads to a quantifiable decrease in FRET signals. The combination of donor and acceptor fluorophore, protein and antibody concentration and incubation times were determined experimentally, with the assay optimised for screening in 384 well plate format using automated liquid handling.

**Figure 2:**
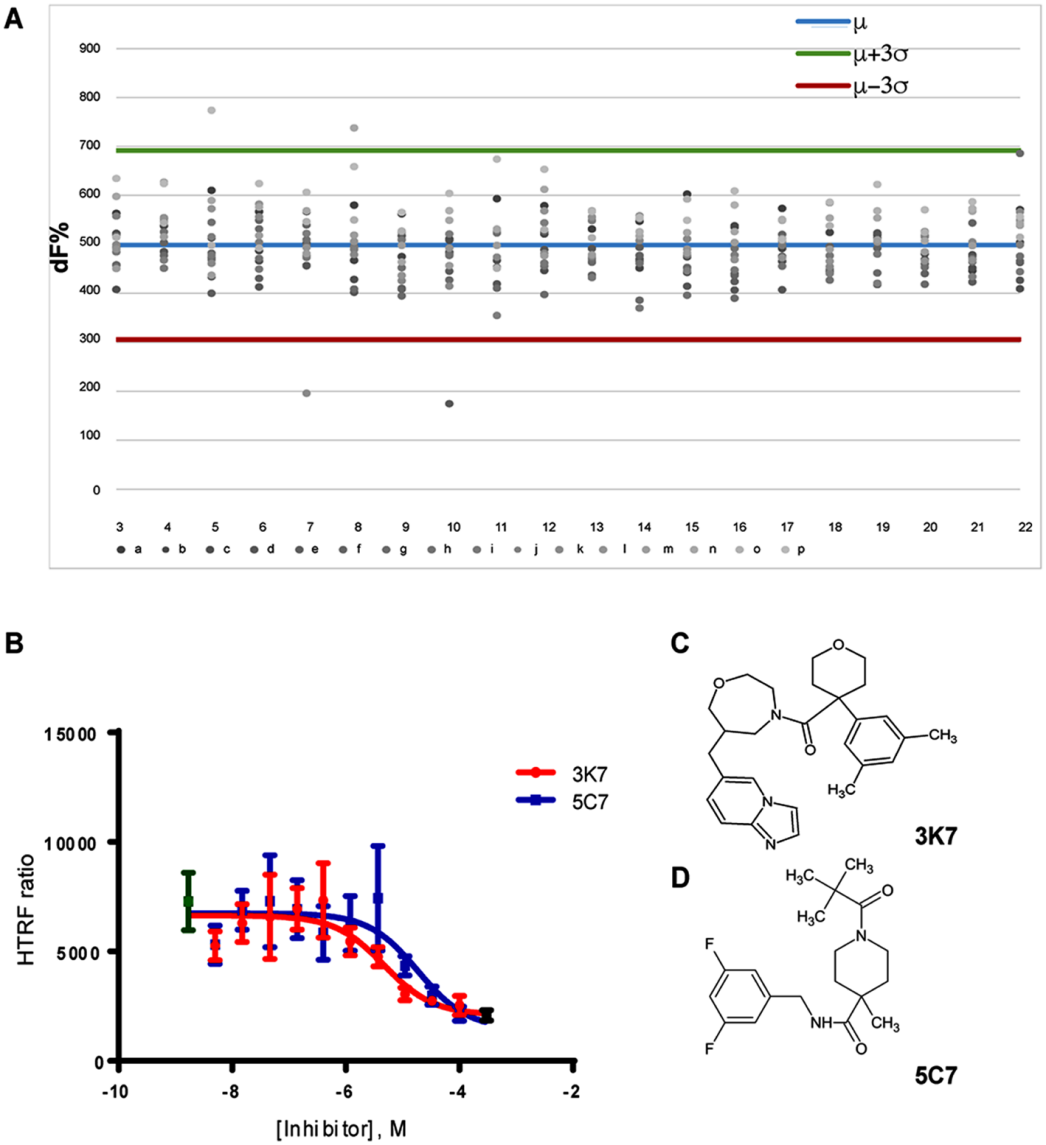
Primary HTRF screen identified dose-dependent inhibitors of the SCL-LMO2 PPI. (**A**) Representative example of the FRET ratio data collected for each 384 well plate. The plate mean (blue line) and three standard deviations above (green line) and below (red line) the mean are indicated. Hits are determined as yielding FRET ratios > 3σ below the plate mean. (**B**) Non-linear regression analysis of FRET inhibition by 3K7 (red) and 5C7 (blue) over a concentration gradient. Curves are the average of three independent replicates. Error bars represent standard deviation. Positive (no SCL, black) and negative (DMSO vector, green) controls are shown. (**C**) Chemical structure of compound 3K7 and (**D**) 5C7.

Subsequently, the HTRF assay was used to screen a library of small molecules for activity as inhibitors of the SCL-LMO2 PPI. To maximise our potential hit rate from screening, the Protein-Protein Interactions Network (PPI-Net) library was used as it contains predominantly large, hydrophobic molecules which are typical of PPI inhibitors. 1534 compounds were screened at a single point concentration of 10 μM, with hits determined as resulting in a reduction in FRET ratio greater than three standard deviations from the plate mean over two independent replicates (Figure 2A). Statistical significance was determined by z’ score calculated using in-plate controls (DMSO vector (negative) and LMO2 only (positive) (Table 1).

**Table 1:** HTRF assay parameters

	Screen 1	Screen 2	Average
**Mean FRET ratio**	9899.57	9628.42	9764.00
**Std deviation**	1077.75	1282.27	1179.51
**CV %**	10.89	13.17	12.03
**dF %**	502.71	354.39	424.05
**z’**	0.55	0.41	0.48
**Total hits**	7	15	11
**Repeated hits**	2	2	2

From the initial screen, two compounds were identified as reproducibly inhibiting the LMO2-SCL PPI with an additional six compounds observed to give a reduced FRET ratio in at least one replicate screen. The HTRF assay was repeated with these eight compounds to determine the dose-response (Supplementary Figure 1). From the initial eight, two compounds, 3K7 and 5C7, (Figure 2C, 2D) resulted in a reproducible, dose-dependent inhibition of the SCL-LMO2 PPI over 3 independent repeats with calculated IC_50_ values of 14.7 μM and 18.7 μM respectively (Figure 2B). The compounds were repurchased from the source and purity and structural identity were confirmed by mass spectrometry and ^1^H NMR (Supplementary Figure 2) before re-testing in the HTRF assay. A similar profile of dose-dependent inhibition (data not shown) was observed, confirming that the identified small molecules act as inhibitors of the SCL-LMO2 PPI *in vitro.*

### 3K7 acts through direct and specific interaction with LMO2

**Figure 3:**
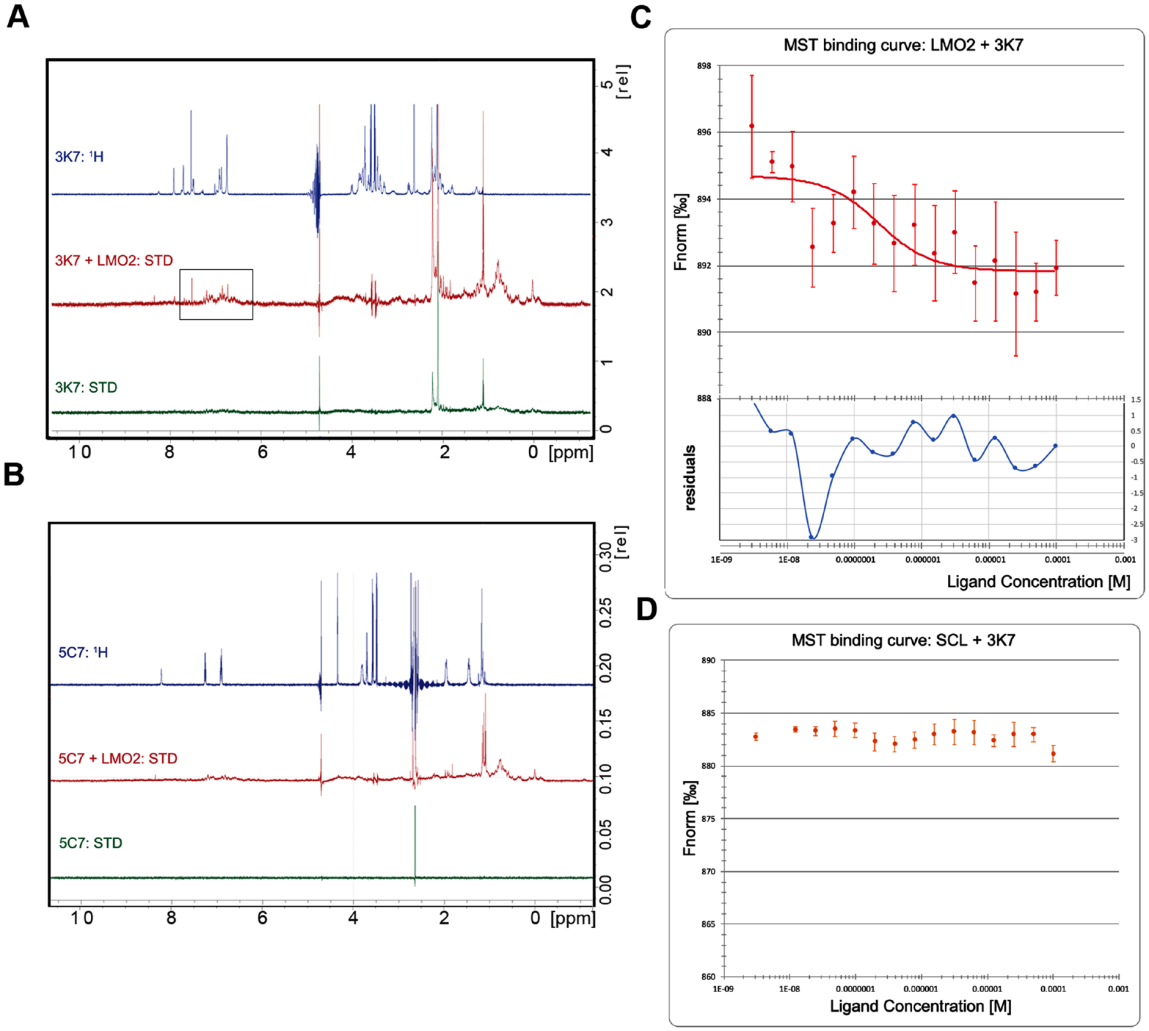
3K7 binds directly to LMO2. STD-NMR spectra for compound alone (green) or in complex with LMO2 (red) were compared to reference ^1^H spectra (blue) for (**A**) 3K7 and (**B**) 5C7. Box highlights peaks indicative of binding observed in the aromatic region of the 3K7 spectrum. (**C**) Curve showing normalised fluorescence data from MST experiments with LMO2 and (**D**) SCL/E47 in presence of increasing 3K7 concentration. The bottom panel in C shows residuals between the data and the fit line. Error bars represent standard deviation, *n* = 4.

To determine the mechanism of inhibition, we investigated the binding of 3K7 and 5C7 to LMO2 and SCL/E47 using ligand-observed STD-NMR [33, 34]. Peaks corresponding to the ^1^H spectrum of 3K7 were observed in the collected STD-NMR spectrum in the presence of LMO2, indicative of binding (Figure 3A). In comparison, no binding of compound 5C7 to LMO2 was detected in these experiments (Figure 3B). To further investigate the mode of interaction of 3K7 and 5C7, we used microscale thermophoresis (MST) [35, 36]. Purified proteins were labelled on free amine residues with a NHS-647 dye (Nanotemper), and thermophoresis of labelled proteins was monitored in the presence of increasing concentrations of inhibitor. Analysis of the data revealed a dose-dependent interaction of 3K7 with LMO2, with a calculated Kd of 1.2 μM (Figure 3C). No clear binding was observed with SCL, suggesting that the mechanism of inhibition is through a direct interaction with LMO2 (Figure 3D). In contrast, compound 5C7 was observed to induce dose-dependent changes in the protein fluorescence, likely caused by non-specific binding or compound-induced aggregation of the proteins (Supplementary Figure 3). From these data, we conclude that the identified SCL-LMO2 PPI inhibitor, 3K7, acts through a direct interaction with LMO2.

In further experiments we addressed the specificity of the 3K7 interaction with LMO2. MST experiments were repeated to determine the affinity of 3K7 for the other 3 known members of the LMO family: LMO1, LMO3 and LMO4. LMO2 shares ~50% sequence homology with LMO1 and LMO3, and < 40% with LMO4. The crystal structures of LMO2 [28, 31] and LMO4 [37, 38] showed strong structural homology of the individual LIM domains (128 residues superimposing within an RMSD of 2.7 Å) and more extensive structural homology is expected between LMO2, LMO1 and LMO3. From the functional point of view, LMO1, LMO2 and LMO3 have been associated with haematopoiesis and T-ALL, whilst LMO4 is functionally more divergent.

**Figure 4:**
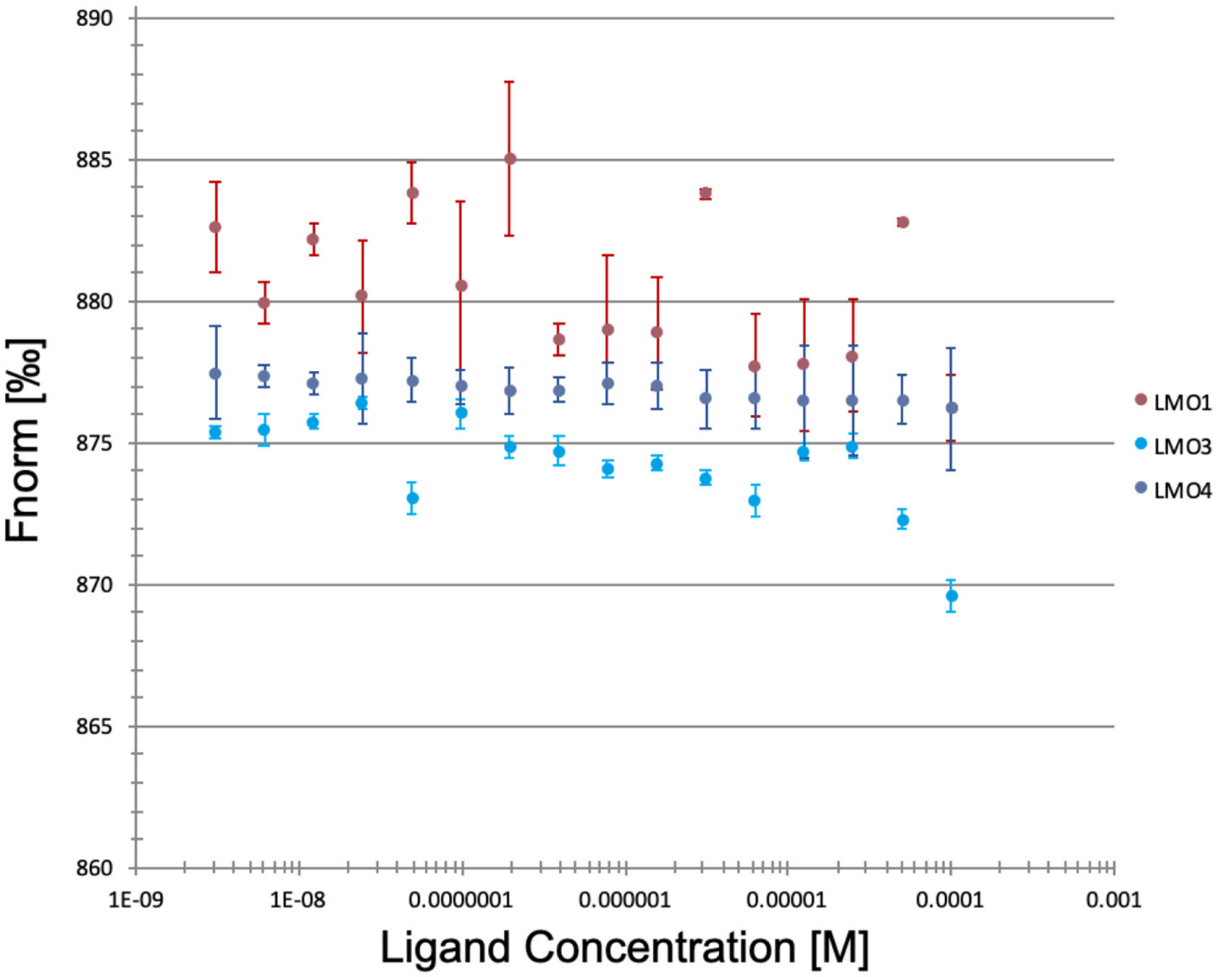
3K7 does not bind to other LMO family proteins. Curves showing normalised fluorescence data from MST experiments looking at 3K7 binding to LMO1 (pink), LMO3 (blue), LMO4 (violet). Error bars represent standard deviation, *n* = 3.

The MST analysis showed no interaction between 3K7 and LMO1, LMO3 or LMO4 (Figure 4). Taken together our data indicate that 3K7 forms a direct and specific interaction with LMO2.

### 3K7 induced conformational change in LMO2 comparable to SCL-binding deficient mutant

To further elucidate the potential mechanism of 3K7-mediated inhibition of the SCL-LMO2 interaction, we set out to investigate the impact of 3K7 binding on the conformational flexibility of LMO2.

**Figure 5:**
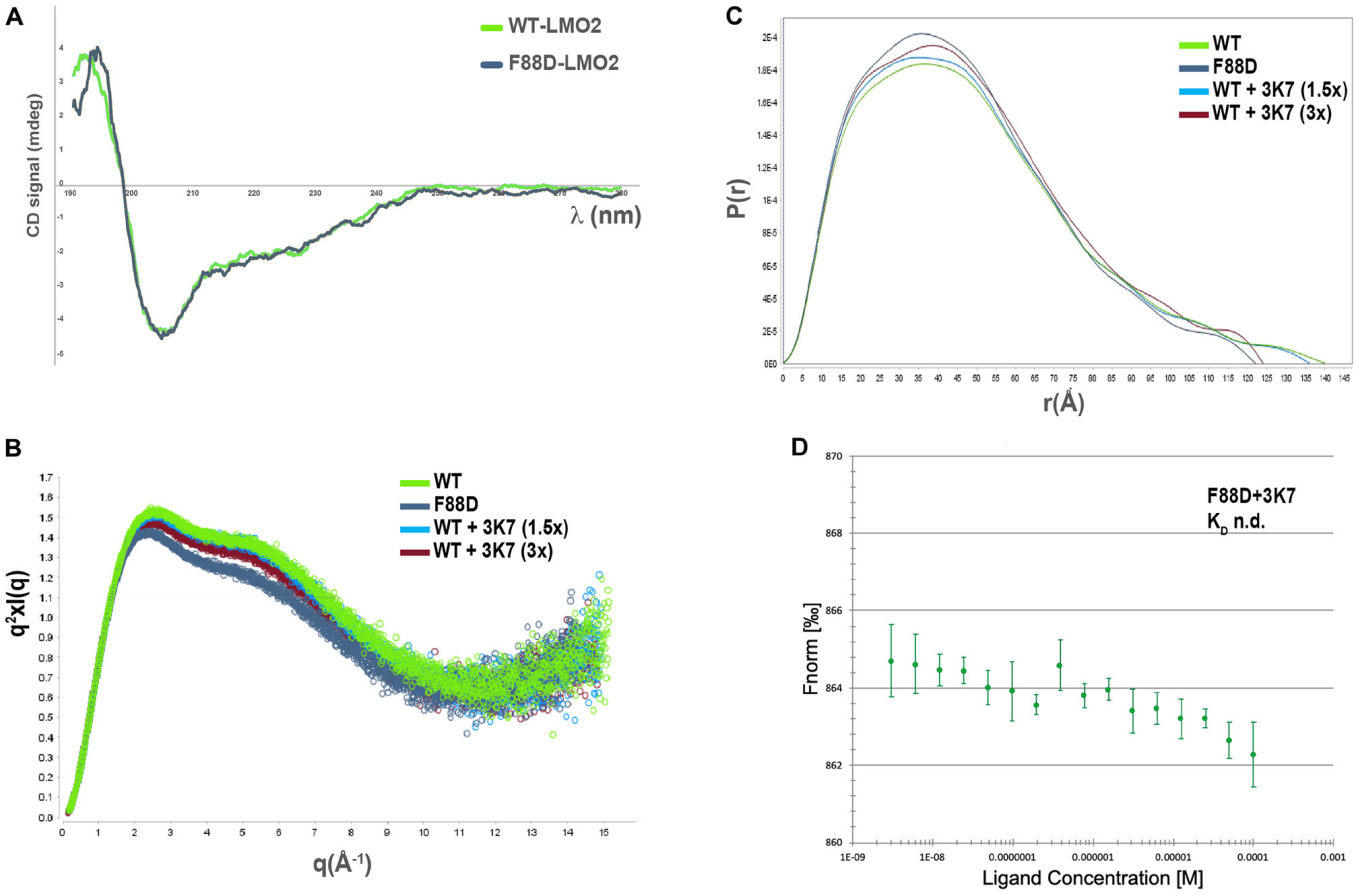
3K7 induces a change in LMO2 conformation comparable to LMO2-F88D. (**A**) Comparison of the far-UV CD spectra for LMO2 (green) and F88D (gray) shows profiles consistent with folded proteins containing similar secondary structures elements. (**B**) Kratky plot of the solution scattering showing broad bell-shaped curves typical of elongated, flexible protein molecules (green: LMO2; gray: LMO2-F88D; light blue: LMO2+ 1.5x 3K7; red: LMO2+3x 3K7). (**C**) Overlay of the P(r) distribution curves characterizing LMO2 (green), LMO2-F88D (gray), and LMO2 incubated with 1.5× (light blue) and 3× (red) molar concentrations of 3K7. The goodness of the data fitting was assessed by calculation of the χ^2^ value, with best fit approximating to 1. The shape of curves is characteristic for elongated molecules. (**D**) Curve showing normalised fluorescence data from MST experiments with LMO2-F88D with increasing 3K7 concentrations. Error bars represent standard deviation, *n* = 3.

Previously published crystallography data [16, 28] revealed large movements around a conserved hinge (F88) between the LIM domains. Mutation of the hinge residue (F88D) demonstrated that this residue is absolutely required for binding of LMO2 to its partner protein SCL/TAL1 *in vitro* and for the function of this complex *in vivo.* As this residue is located in proximity of the SCL interface, it is possible that mutation of this residue disrupts the binding surface. Another possibility is that a mutation in the hinge region affects the accessible conformations of the proteins. The effect of the F88D mutation on LMO2 conformation was explored using small angle X-ray scattering (SAXS) to observe the protein in solution [39, 40]. Firstly, circular dichroism spectroscopy (CD) determined that the F88D is soluble and correctly folded with no significant deviation observed from the WT profile suggesting no changes in the secondary structure (Figure 5A). Next, WT and F88D were subjected to SEC-SAXS to obtain information on the shape and the size of these proteins. By using a Kratky representation to evaluate the globularity and flexibility, we observe that LMO2 and F88D have similar scattering profiles and are multidomain proteins connected by a flexible linker (Figure 5B). Analysis of the pair-wise distance distribution function P(r) however, showed a reduced in the maximum distance (D_max_) of F88D (Figure 5C) and of the calculated radius of gyration (R_g_) (Table 2) when comparing F88D to LMO2, suggesting that the mutant protein on average adopts a more constrained conformation. The data therefore suggests that the F88D mutation causes modulation of the LMO2 conformational flexibility.

**Table 2:** Structural parameters from SAXS

	Lmo2	LMO2-F88D	LMO2+3K7 (1.5)	LMO2+3K7 (3)
**R_g_ (Å)**	38.85	36.56	38.32	38.03
**D_max_ (Å)**	140	122	136	124
**χ^2^**	1.06	1.47	0.98	0.89

We hypothesised that 3K7 exercises its inhibitory effect through a similar mechanism of conformational modulation. Addition of increasing concentrations of 3K7 to WT prior to the SAXS experiments resulted in a shift of the protein Dmax (Figure 5C) and R_g_ (Table 2) to values similar to those of F88D, suggesting that in solution the proteins adopts a constrained conformation, similar to that of F88D. Next, we hypothesised that 3K7 is a conformation-specific ligand, binding to LMO2 only in its SCL-binding competent form. To test this hypothesis, we investigated binding of 3K7 to F88D. Over multiple repeats, no evidence of compound binding was observed (Figure 5D), supporting the suggestion that the conformational state adopted by LMO2 following the F88D mutation is not compatible with 3K7 binding.

## DISCUSSION

Protein-protein interactions are challenging, yet potentially valuable targets for drug discovery. A particular attractive strategy in the quest to design novel anticancer agents is to target transcription factors PPIs, as TF operate at junction points in most oncogenic signalling pathways and are often functionally altered in many cancers. Targeting PPIs by small molecule inhibitors however has historically been considered a challenging undertaking. Large surface areas and the lack of pockets at the PPI interface have elicited the long-held belief that this class of interactions is “undruggable”. The paradigm is however shifting, in part thanks to recent advances in biophysical and structural techniques, which have provided clearer insights into PPI interactions. These studies have clearly established that the bulk of the binding energy at PPI interfaces is provided only by a small subset of amino acids and that targeting these “hot spots” is likely to destabilise the interaction. An ever-increasing number of success stories of small molecules modulators of PPI bodes well for structure-based approaches to this [41–43].

When considering inhibiting transcription factors’ PPIs, two main approaches have been explored: disrupting (homo or hetero) dimerization, or targeting interactions with other transcriptional cofactors. Whilst the first approach is likely to abolish function by blocking DNA binding, the second has the potential to modulate function by inhibiting potential oncogenic interactions.

In T-ALL, ectopically expressed transcription factor SCL interacts with transcriptional cofactor LMO2 to prevent the activity of E protein homodimers, essential for normal progression of T-cell differentiation. Detailed analysis of the SCL-LMO2 PPI interface, as revealed by the crystal structure of the complex, uncovered a small interface area (620 Å^2^) suggesting that the bulk of the binding energy is provided only by a small subset of amino acids around the hinge domains of LMO2 and that targeting this “hot spot” by small molecules is likely to destabilise this T-ALL oncogenic interaction.

To find small molecules capable of disrupting the SCL-LMO2 interaction we used HTRF as a primary screen. Labelling proteins with fluorophores conjugated to affinity antibodies allows for monitoring of complex formation and subsequent FRET emission, without compromise of the interaction interface when compared to covalent modification of the proteins. HTRF can be easily optimised for automated liquid handling allowing rapid screening of large compound libraries. Use of a specific PPI library designed to target larger, hydrophobic interfaces provided an increased probability of identifying hits. From the primary screens, two molecules were identified as dose-responsive SCL-LMO2 PPI inhibitors, an acceptable 0.13% hit rate, following hit reconfirmation (purity and structure confirmation). However, following these encouraging biochemical screening data, it was essential to provide confirmatory, orthogonal evidence of disruption of the SCL-LMO2 interaction.

One of these compounds, 3K7, was found to be directly and specifically binding to LMO2 but not to other members of the LMO family. The low solubility of the 3K7 meant that further structural biology techniques (X-ray crystallography, NMR) aimed at ascertaining the molecular details of its interaction with LMO2 were not successful. By using SAXS, we were able to demonstrate that 3K7 appears to modulate the conformational flexibility of LMO2, with the protein adopting a less extended conformation. Previous work uncovered substantial conformational flexibility of LMO2 around the conserved hinge between the LIM domains which proved necessary for binding SCL. Specifically, mutation of F88 in the hinge domain induced a conformational change in LMO2 which prevented it from binding to SCL. Our SAXS data suggest that 3K7 binding to LMO2 alters the conformational flexibility of LMO2 in a comparable way to the F88D mutation, leading us to speculate that conformational modulation is the mechanism of action of this small molecule drug.

Our MST data shows that 3K7 has little affinity for other member of the LMO family, implying that 3K7 is LMO2 specific. Structural analysis of the atomic structures had previously suggested that despite highly homologous secondary structure arrangements, widely different conformational states were adopted by LMO2 and LMO4 around their hinge domains. Sequence divergence of LMO proteins is likely to be responsible for the alternative conformational states, thus regulating the varied PPIs they are thought to be mediating. This provides a potential explanation for the specificity of 3K7 for LMO2 over the closely related LMO proteins and further could allow for specific targeting. This hypothesis is further supported by the observation that 3K7 is unable to bind to the F88D LMO2 mutant, which like LMO4 adopts a distinct subset of conformations when compared to WT LMO2.

The low aqueous solubility of 3K7 prevented additional cellular assays to be performed, restricting the prospect of testing this compound *in vivo*. Low solubility also prevented a more in depth structural analysis of the binding mode of 3K7, limiting further chemical development of the compound. Nonetheless, our data provide evidence that the effect of small molecules on modulating protein conformation can be potentially used as a powerful approach to drug discovery, even for otherwise intractable PPIs. Specifically, as protein conformation is key to mediating PPIs between flexible, minimally structured proteins, future work could exploit this reliance on conformational flexibility to engineer specific therapeutics that target transcription factors.

However, we posit that not all PPIs are equally tractable for blocking by small molecules that modulate conformational flexibility and detailed analysis of PPI interfaces is critical for selection of those with the highest chance of success.

## MATERIALS AND METHODS

### Materials

A library of 1534 compounds was obtained from PPI-net. Additional compounds were purchased from suppliers (3K7 – Asinex, 5C7 – ChemDiv).

### Expression constructs

A tandem LIM-domain construct of LMO2 fused to the LDB1^LID^ (FLINC2, [31]) was used for CD spectroscopy and MST. The protein was subcloned into a pET47b vector containing a HRV 3C cleavable N-terminal 6x-His tag by Gibson Assembly to allow production of untagged protein for NMR and SAXS (pET47-LMO2), and subsequently a C-terminal FLAG tag was introduced by PCR for HTRF (LMO2-FLAG).

The F88D mutation was introduced into each construct as required by site-directed mutagenesis using standard PCR protocols [16].

The bHLH domains of SCL and E47 are co-expressed from one construct (pETDuet-E47/SCL, [16]). An N-terminal Strep-II tag was introduced to the SCL sequence by PCR.

Tandem-LIM domain constructs of LMO1 (aa 20-149), LMO3 (aa 9-139) fused to the LDB1^LID^ (aa 336-375) in the pETDuet vector were used for MST as specified in the results. A tandem-LIM construct of LMO4 (aa 16-152, including C52S/C64S mutations) fused to the LDB1^LID^ (aa 336-375) [37] in the pET47b(+) vector was also used for MST as specified.

### Protein expression and purification

LMO2 proteins were expressed in *E.coli* and purified as previously published [31]. For HTRF experiments, cleavable His-tags were removed by overnight incubation at 4°C with HRV-3C protease prior to SEC purification. Other LMO proteins were expressed and purified as LMO2.

SCL protein was co-expressed with E47 in *E.coli* as previously published [16]. Initial purification on cobalt resin (Generon) in IMAC buffer (50 mM sodium phosphate pH 8, 500 mM NaCl, 0.5 mM TCEP) eluting in 300 mM imidazole, followed by purification on StrepTactin (GE Healthcare) eluting in 2.5 mM desthiobiotin (Sigma) yielded isolated His-E47/Strep-SCL heterodimers. Additional SEC purification using Superdex75 columns (GE Healthcare) was carried out as required in SEC buffer (20 mM HEPES pH 7.4, 200 mM NaCl, 0.5 mM TCEP).

### Homogeneous Time-Resolved Fluorescence (HTRF)

Small molecules from a 1534 compound library (PPI-net) were transferred to 384 well plates (2 μL each 50 μM stock in HTRF buffer (25 mM HEPES pH 7.5, 250 mM NaCl) plus 5% DMSO, final concentration 10 μM) using a FluidX XPP-721 automated liquid handling system. Purified LMO2-FLAG and His-E47/Strep-SCL proteins were diluted to 250 nM in HTRF buffer and mixed 1:1 immediately prior to use. 8 μL mixed proteins were added to each well to a final concentration of 100 nM using a Multidrop Combi reagent dispenser (Thermo Fisher Scientific) and plates were incubated at room temperature for 2 hours at room temperature. Control wells (maximum FRET: mixed proteins + DMSO; no FRET: LMO2 + DMSO) were included on each plate. Fluorophore-conjugated affinity antibody stocks (anti-FLAG terbium cryptate, anti-His-d2, CisBio) were diluted 1:100 in HTRF buffer before mixing 1:1 and adding to 384 well plates (10 μL/well, Multidrop Combi). Plates were incubated for a further 2 hours at room temperature before detecting FRET signals using a PHERAstar FS (BMG LabTech) plate reader with HTRF 337/620/655 nm optic module installed. Excitation at 337 nm was carried out using an integration delay of 60 μsec, integration time 400 μsec, 40 flashes (laser), with an automatically determined focal height. Emissions at 620 and 655 nm were measured to calculate the FRET ratio dF%. Hits were determined as giving a reduction in FRET ratio > 3 SD from plate mean over 2 independent repeats. Significance was determined by calculating z’ score for each plate/data set [44]. To check for the effect of DMSO, FRET ratio dF% was measured by mixing 50 nM of each protein and 5 μL of each antibodies with increasing concentrations of DMSO (0, 1, 2, 5, 10% DMSO) and reading the plate at 0, 1, 2 and 3 hours after addition of the antibodies. A decrease in HTRF signal with the addition of any percentage of DMSO was recorded, but no significant difference in signal with concentrations between 1–10% at each time point (Supplementary Figure 4). Dose-response of selected compounds was determined over 10 3-fold serial dilutions from 100 μM. Data from three independent repeats was analysed by non-linear regression (GraphPad Prism), error bars represent standard deviation from 3 independent repeats.

### Microscale Thermophoresis (MST)

Purified proteins were individually labelled with fluorescent dye NT647 using a Monolith NT_TM_ Protein Labeling Kit (NanoTemper Technologies). Serial dilutions of 3K7 and 5C7 (100 μM–6 nM) in MST buffer (50 mM Tris-HCl pH 7.8, 150 mM NaCl, 10 mM MgCl_2_, 0.05% Tween 20, 2% DMSO) were mixed with 100 nM NT647-labeled protein, incubated for 15 minutes at room temperature and loaded into standard glass capillaries (Monolith NT.115 Capillaries, NanoTemper Technologies). Thermophoresis analysis was performed over 30 seconds on a Monolith NT.115 instrument (20% LED, 20/40% MST power) at 22°C. The MST curves were fitted using NT Analysis software (NanoTemper Technologies) to obtain Kd values for binding.

For SD-tests, MST samples were mixed 1:1 with 2X SD mix (4% SDS, 40 mM DTT) and incubated at 95 °C for 5 minutes to denature. Following centrifugation, samples were loaded into standard glass capillaries and the fluorescence intensity was re-tested.

### Saturation-Transfer Difference NMR (STD-NMR)

STD-NMR experiments [34] were performed on a Bruker Advance III 900 MHz spectrometer equipped with a 5 mm cryogenic probe at 25ºC. Samples contained 100 mM compounds and 5 μM LMO2 protein. Selective saturation of the protein was achieved using a train of Gaussian pulses at –0.5 ppm for a total saturation time of 3s. Off-resonance irradiation was set at 30 ppm. Data were collected using 32k complex points in the direct dimension. 512 scans were collected for each experiment and a interscan delay of 3 seconds was used to allow relaxation of the sample. Water suppression was achieved using excitation sculpting.

### CD spectroscopy

Far-UV CD analysis of LMO2 and LMO2-F88D proteins was performed on a Jasco J715 Spectropolarimeter. The spectra were recorded over wavelength range 190–280 nm in a 0.1 mm cell at sample concentration of 30 μM at 21°C in CD buffer (20 mM HEPES pH 7.5, 100 mM NaF). The final spectra were the average of 3 scans. CD spectra of the buffer solutions in the appropriate cuvette were subtracted from the sample spectra and smoothed using the Savistky–Golay function before conversion to absolute CD values. Curves were generated in Excel.

### Small Angle X-ray Scattering (SAXS)

Purified LMO2 and LMO2-F88D proteins were diluted to 40 μM in SAXS buffer (20 mM HEPES pH 7.5, 200 mM NaCl, 0.5 mM TCEP). 3K7 was added as required (1.5 and 3 molar equivalents, 1% DMSO final) before incubation for 1 hour at room temperature. X-ray scattering patterns were recorded from solutions of the individual proteins on beamline B21 at Diamond Light Source. Samples were run through a Shodex KW402.5 column with an Agilent FPLC at 0.16 mL/min and then applied directly into the beam. Scattering was recorded on a Pilatus 2M detector with a 0.2 × 0.2 mm, 12.4 KeV (1 Å) beam. Sample to detector distance set at 4000 mm, exposure time of 3 seconds (10 readings/exposure in HPLC mode) at 293 K. Data processing (background subtraction and radius of gyration (Rg) calculation) was performed using ScÅtter (v3.0 by Robert P. Rambo; Diamond Light Source).

## SUPPLEMENTARY MATERIALS



## References

[1] Porcher C , Chagraoui H , Kristiansen MS . SCL/TAL1: a multi-faceted regulator from blood development to disease. Blood. 2017; 129:2051–2060. 10.1182/blood-2016-12-754051. 28179281

[2] Porcher C , Swat W , Rockwell K , Fujiwara Y , Alt FW , Orkin SH . The T-cell leukemia oncoprotein SCL/tal-1 is essential for development of all haematopoietic lineages. Cell. 1996; 86:47–57. 10.1016/S0092-8674(00)80076-8. 8689686

[3] Wadman IA , Osada H , Grutz GG , Agulnick AD , Westphal H , Forster A , Rabbitts TH . The LIM-only protein Lmo2 is a bridging molecule assembling an erythroid, DNA-binding complex which includes the TAL1, E47, GATA-1 and Ldb1/NLI proteins. EMBO J. 1997; 16:3145–57. 10.1093/emboj/16.11.3145. 9214632 PMC1169933

[4] Chambers J , Rabbitts TH . LMO2 at 25 years: a paradigm of chromosomal translocation proteins. Open Biol. 2015; 5:150062. 10.1098/rsob.150062. 26108219 PMC4632508

[5] Warren AJ , Colledge WH , Carlton MBL , Evans MJ , Smith AJH , Rabbitts TH . The oncogenic cysteine-rich LIM domain protein Rbtn2 is essential for erythroid development. Cell. 1994; 78:45–57. 10.1016/0092-8674(94)90571-1. 8033210

[6] Mukhopadhyay M , Teufel A , Yamashita T , Agulnick AD , Chen L , Downs KM , Schindler A , Grinberg A , Huang SP , Dorward D , Westphal H . Functional ablation of the mouse Ldb1 gene results in severe patterning defects during gastrulation. Development. 2003; 130:495–505. 10.1242/dev.00225. 12490556

[7] Yamada Y , Pannell R , Forster A , Rabbitts TH . The oncogenic LIM-only transcription factor Lmo2 regulates angiogenesis but not vasculogenesis in mice. Proc Natl Acad Sci U S A. 2000; 97:320–324. 10.1073/pnas.97.1.320. 10618416 PMC26661

[8] Yamada Y , Pannell R , Forster A , Rabbitts TH . The LIM-domain protein Lmo2 is a key regulator of tumour angiogenesis: a new anti-angiogenesis drug target. Oncogene. 2002; 21:1309–15. 10.1038/sj.onc.1205285. 11857074

[9] Yamada Y , Warren AJ , Dobson C , Forster A , Pannell R , Rabbitts TH . The T cell leukemia LIM protein Lmo2 is necessary for adult mouse hematopoiesis. Proc Natl Acad Sci U S A. 1998; 95:3890–3895. 10.1073/pnas.95.7.3890. 9520463 PMC19933

[10] Ferrando AA , Neuberg DS , Staunton J , Loh ML , Huard C , Raimondi SC , Behm FG , Pui CH , Downing JR , Gilliland DG , Lander ES , Golub TR , Look AT . Gene expression signatures define novel oncogenic pathways in T cell acute lymphoblastic leukemia. Cancer Cell. 2002; 1:75–87. 10.1016/S1535-6108(02)00018-1. 12086890

[11] Ferrando AA , Herblot S , Palomero T , Hansen M , Hoang T , Fox EA , Look AT . Biallelic transcriptional activation of oncogenic transcription factors in T-cell acute lymphoblastic leukemia. Blood. 2004; 103:1909–11. 10.1182/blood-2003-07-2577. 14604958

[12] Van Vlierberghe P , Ferrando A . The molecular basis of T cell acute lymphoblastic leukemia. J Clin Invest. 2012; 122:3398–406. 10.1172/JCI61269. 23023710 PMC3461904

[13] Van Vlierberghe P , Beverloo HB , Buijs-Gladdines J , van Wering ER , Horstmann M , Pieters R , Meijerink JP . Monoallelic or biallelic LMO2 expression in relation to the LMO2 rearrangement status in pediatric T-cell acute lymphoblastic leukemia. Leukemia. 2008; 22:1434–7. 10.1038/sj.leu.2405063. 18079736

[14] Matthews JM , Lester K , Joseph S , Curtis DJ . LIM-domain-only proteins in cancer. Nat Rev Cancer. 2013; 13:111–122. 10.1038/nrc3418. 23303138

[15] Lecuyer E , Hoang T . From the origin of haematopoiesis to stem cells and leukemia. Exp Hematol. 2004; 32:11–24. 10.1016/j.exphem.2003.10.010. 14725896

[16] El Omari K , Hoosdally SJ , Tuladhar K , Karia D , Hall-Ponsele E , Platonova O , Vyas P , Patient R , Porcher C , Mancini EJ . Structural basis for LMO2-driven recruitment of the SCL:E47bHLH heterodimer to hematopoietic-specific transcriptional targets. Cell Rep. 2013; 4:135–47. 10.1016/j.celrep.2013.06.008. 23831025 PMC3714592

[17] Schuh AH , Tipping AJ , Clark AJ , Hamlett I , Guyot B , Iborra FJ , Rodriguez P , Strouboulis J , Enver T , Vyas P , Porcher C . ETO-2 associates with SCL in erythroid cells and megakaryocytes and provides repressor functions in erythropoiesis. Mol Cell Biol. 2005; 25:10235–50. 10.1128/MCB.25.23.10235-10250.2005. 16287841 PMC1291220

[18] Rahman S , Magnussen M , Leon TE , Farah N , Li Z , Abraham BJ , Alapi KZ , Mitchell RJ , Naughton T , Fielding AK , Pizzey A , Bustraan S , Allen C , et al. Activation of the LMO2 oncogene through a somatically acquired neomorphic promoter in T-cell acute lymphoblastic leukemia. Blood. 2017; 129:3221–3226. 10.1182/blood-2016-09-742148. 28270453 PMC5472898

[19] Howe SJ , Mansour MR , Schwarzwaelder K , Bartholomae C , Hubank M , Kempski H , Brugman MH , Pike-Overzet K , Chatters SJ , de Ridder D , Gilmour KC , Adams S , Thornhill SI , et al. Insertional mutagenesis combined with acquired somatic mutations causes leukemogenesis following gene therapy of SCID-X1 patients. J Clin Invest. 2008; 118:3143–50. 10.1172/JCI35798. 18688286 PMC2496964

[20] Tremblay CS , Curtis DJ . The clonal evolution of leukemic stem cells in T-cell acute lymphoblastic leukemia. Curr Opin Hematol. 2014; 21:320–325. 10.1097/MOH.0000000000000058. 24857886

[21] Aplan PD , Jones CA , Chervinsky DS , Zhao XF , Ellsworth M , Wu C , McGuire EA , Gross KW . An *scl* gene product lacking the tranactivation domain induces bony abnormalities and cooperates with LMO1 to generate T-cell malignancies in transgenic mice. EMBO J. 1997; 16:2408–2419. 10.1093/emboj/16.9.2408. 9171354 PMC1169841

[22] Tremblay M , Tremblay CS , Herblot S , Aplan PD , Hebert J , Perreault C , Hoang T . Modeling T-cell acute lymphoblastic leukemia induced by the SCL and LMO1 oncogenes. Genes Dev. 2010; 24:1093–105. 10.1101/gad.1897910. 20516195 PMC2878648

[23] Herblot S , Steff AM , Hugo P , Aplan PD , Hoang T . SCL and LMO1 alter thymocyte differentiation: inhibition of E2A-HEB function and pre-Talpha chain expression. Nat Immunol. 2000; 1:138–44. 10.1038/77819. 11248806

[24] Chervinsky DS , Zhao XF , Lam DH , Ellsworth M , Gross KW , Aplan PD . Disordered T-cell development and T-cell malignancies in SCL LMO2 double-transgenic mice: parallels with E2A-deficient mice. Mol Cell Biol. 1999; 19:5025–35. 10.1128/MCB.19.7.5025. 10373552 PMC84335

[25] Larson RC , Lavenir I , Larson TA , Baer R , Warren AJ , Wadman I , Nottage K , Rabbitts TH . Protein dimerization between Lmo2 (Rtbn2) and Tal1 alters thymocyte development and potentiates T cell tumorigenesis in transgenic mice. EMBO J. 1996; 15:1021–7. 10.1002/j.1460-2075.1996.tb00439.x. 8605871 PMC449997

[26] Ferrando AA . The role of NOTCH1 signaling in T-ALL. Hematology Am Soc Hematol Educ Program. 2009; 2009:353–61. 10.1182/asheducation-2009.1.353. 20008221 PMC2847371

[27] Ryan DP , Duncan JL , Lee C , Kuchel PW , Matthews JM . Assembly of the oncogenic DNA-binding complex LMO2-Ldb1-TAL1-E12. Proteins. 2008; 70:1461–74. 10.1002/prot.21638. 17910069

[28] El Omari K , Hoosdally SJ , Tuladhar K , Karia D , Vyas P , Patient R , Porcher C , Mancini EJ . Structure of the leukemia oncogene LMO2: implications for the assembly of a hematopoietic transcription factor complex. Blood. 2011; 117:2146–56. 10.1182/blood-2010-07-293357. 21076045

[29] Sewell H , Tanaka T , El Omari K , Mancini EJ , Cruz A , Fernandez-Fuentes N , Chambers J , Rabbitts TH . Conformational flexibility of the oncogenic protein LMO2 primes the formation of the multi-protein transcription complex. Sci Rep. 2014; 4:3643. 10.1038/srep03643. 24407558 PMC3887373

[30] Degorce F , Card A , Soh S , Trinquet E , Knapik GP , Xie B . HTRF: A technology tailored for drug discovery - a review of theoretical aspects and recent applications. Curr Chem Genomics. 2009; 3:22–32. 10.2174/1875397300903010022. 20161833 PMC2802762

[31] El Omari K , Porcher C , Mancini EJ . Purification, crystallization and preliminary X-ray analysis of a fusion of the LIM domains of LMO2 and the LID domain of Ldb1. Acta Crystallogr Sect F Struct Biol Cryst Commun. 2010; 66:1466–9. 10.1107/S1744309110032872. 21045296 PMC3001649

[32] Deane JE , Sum E , Mackay JP , Lindeman GJ , Visvader JE , Matthews JM . Design, production and characterization of FLIN2 and FLIN4: the engineering of intramolecular ldb1:LMO complexes. Protein Eng. 2001; 14:493–9. 10.1093/protein/14.7.493. 11522923

[33] Ciulli A . Biophysical screening for the discovery of small-molecule ligands. Methods Mol Biol. 2013; 1008:357–388. 10.1007/978-1-62703-398-5_13. 23729259 PMC4441727

[34] Mayer M , Meyer B . Characterization of Ligand Binding by Saturation Transfer Difference NMR Spectroscopy. Angew Chem Int Ed Engl. 1999; 38:1784–1788. 10.1002/(SICI)1521-3773(19990614)38:12<1784::AID-ANIE1784>3.0.CO;2-Q. 29711196

[35] Jerabek-Willemsen M , Wienken CJ , Braun D , Baaske P , Duhr S . Molecular interaction studies using microscale thermophoresis. Assay Drug Dev Technol. 2011; 9:342–53. 10.1089/adt.2011.0380. 21812660 PMC3148787

[36] Duhr S , Braun D . Why molecules move along a temperature gradient. Proc Natl Acad Sci U S A. 2006; 103:19678–19682. 10.1073/pnas.0603873103. 17164337 PMC1750914

[37] Deane JE , Maher MJ , Langley DB , Graham SC , Visvader JE , Guss JM , Matthews JM . Crystallization of FLINC4, an intramolecular LMO4-ldb1 complex. Acta Crystallogr D Biol Crystallogr. 2003; 59:1484–6. 10.1107/S0907444903011843. 12876360

[38] Deane JE , Ryan DP , Sunde M , Maher MJ , Guss JM , Visvader JE , Matthews JM . Tandem LIM domains provide synergistic binding in the LMO4:Ldb1 complex. EMBO J. 2004; 23:3589–98. 10.1038/sj.emboj.7600376. 15343268 PMC517615

[39] Mertens HD , Svergun DI . Structural characterization of proteins and complexes using small-angle X-ray solution scattering. J Struct Biol. 2010; 172:128–141. 10.1016/j.jsb.2010.06.012. 20558299

[40] Vestergaard B . Analysis of biostructural changes, dynamics, and interactions - Small-angle X-ray scattering to the rescue. Arch Biochem Biophys. 2016; 602:69–79. 10.1016/j.abb.2016.02.029. 26945933

[41] Arkin MR , Tang Y , Wells JA . Small-molecule inhibitors of protein-protein interactions: progressing towards the reality. Chem Biol. 2014; 21:1102–14. 10.1016/j.chembiol.2014.09.001. 25237857 PMC4179228

[42] Arkin MR , Whitty A . The road less traveled: modulating signal transduction enzymes by inhibiting their protein-protein interactions. Curr Opin Chem Biol. 2009; 13:284–90. 10.1016/j.cbpa.2009.05.125. 19553156

[43] Laraia L , McKenzie G , Spring DR , Venkitaraman AR , Huggins DJ . Overcoming Chemical, Biological, and Computational Challenges in the Development of Inhibitors Targeting Protein-Protein Interactions. Chem Biol. 2015; 22:689–703. 10.1016/j.chembiol.2015.04.019. 26091166 PMC4518475

[44] Zhang JH , Chung TD , Oldenburg KR . A Simple Statistical Parameter for Use in Evaluation and Validation of High Throughput Screening Assays. J Biomol Screen. 1999; 4:67–73. 10.1177/108705719900400206. 10838414

