# Supplementary material for: Small molecule inhibits T-cell acute lymphoblastic leukaemia oncogenic interaction through conformational modulation of LMO2
Source: Oncotarget. 2020 May 12;11(19):1737–48. doi: 10.18632/oncotarget.27580 (PMC7233811; doi:10.18632/oncotarget.27580)
Supplement: Supplementary file 1 [file oncotarget-11-1737-s001.pdf]

Small molecule inhibits T-cell acute lymphoblastic leukaemia oncogenic interaction through conformational modulation of LMO2

SUPPLEMENTARY MATERIALS

A

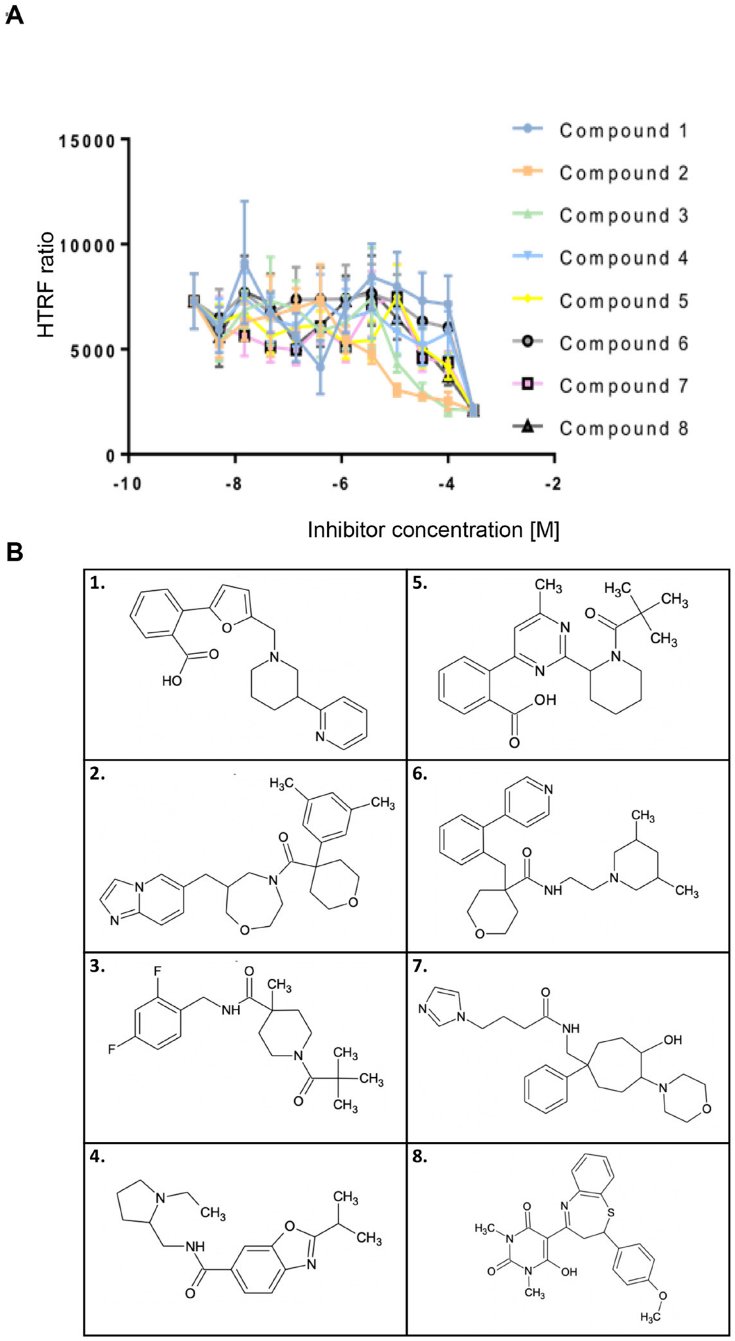

Supplementary Figure 1: HTRF screen identified dose-dependent inhibitors of the SCL-LMO2 PPI. (**A**) Non-linear regression analysis of FRET inhibition by compounds identified as LMO2-SCL PPI inhibitions in an initial HTRF screen, tested over a concentration gradient. Curves are the average of three independent replicates. Error bars represent standard deviation. (**B**) Structures of the 8 compound hits tested in HTRF dose-response assays.

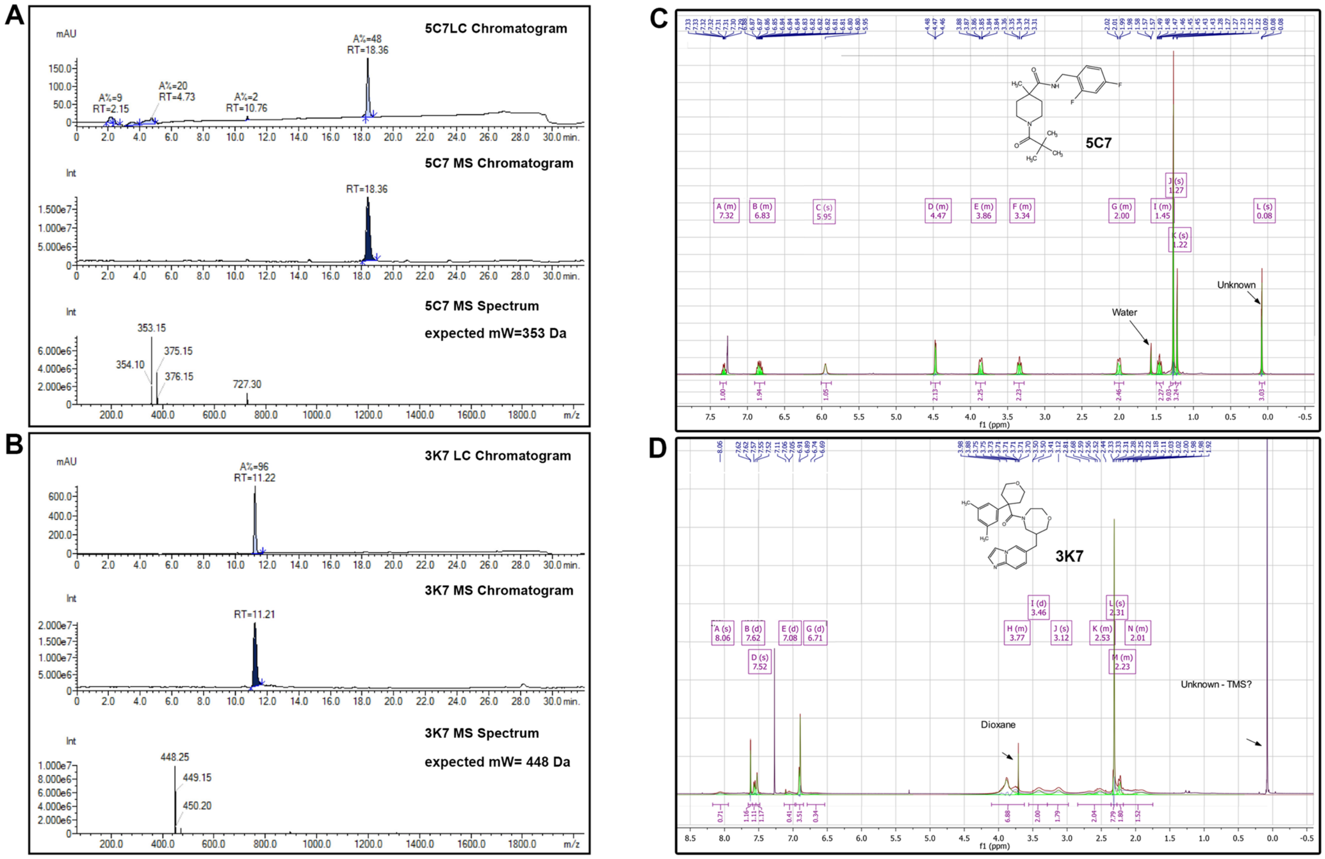

Supplementary Figure 2: Structure of inhibitors 3K7 and 5C7 was validated by mass spectrometry and ^{1}H NMR. LC chromatograms and MS spectra for (**A**) 5C7 and (**B**) 3K7 show fragments of the expected molecular weight. 400 MHz ^{1}H NMR spectrum of (**C**) 5C7 and (**D**) 3K7.

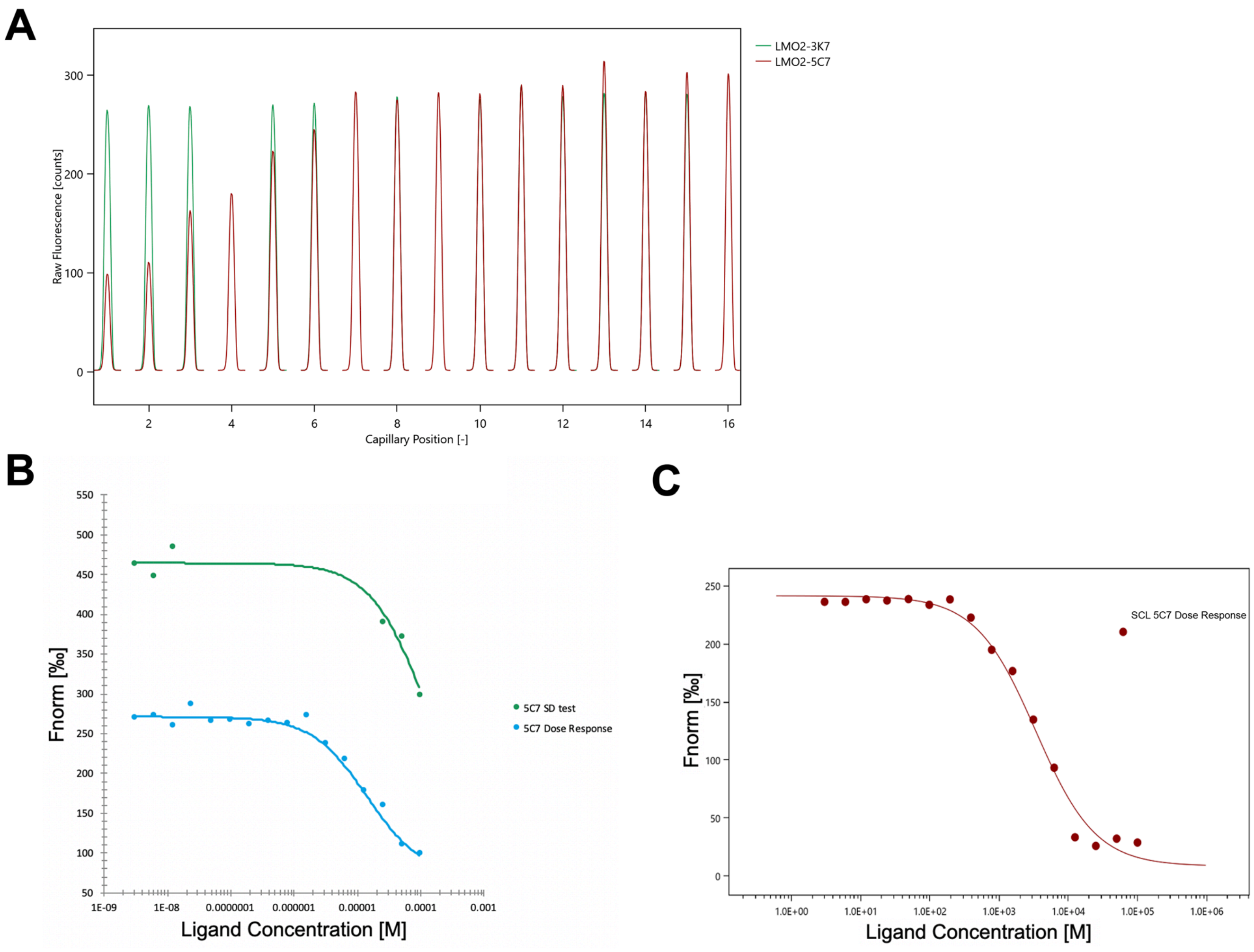

Supplementary Figure 3: 5C7 induces non-specific changes in the MST profile of LMO2 and SCL. (**A**) Fluorescence intensity scan of capillaries containing increasing concentrations of 3C7 and 5C7 (concentration increases right to left). (**B**) Curves showing normalised fluorescence dose response data from MST experiments with LMO2 (with (SD test) or without denaturation) and increasing concentrations of 5C7. (**C**) Curve showing normalised fluorescence data from MST experiments with SCL and increasing concentrations of 5C7.

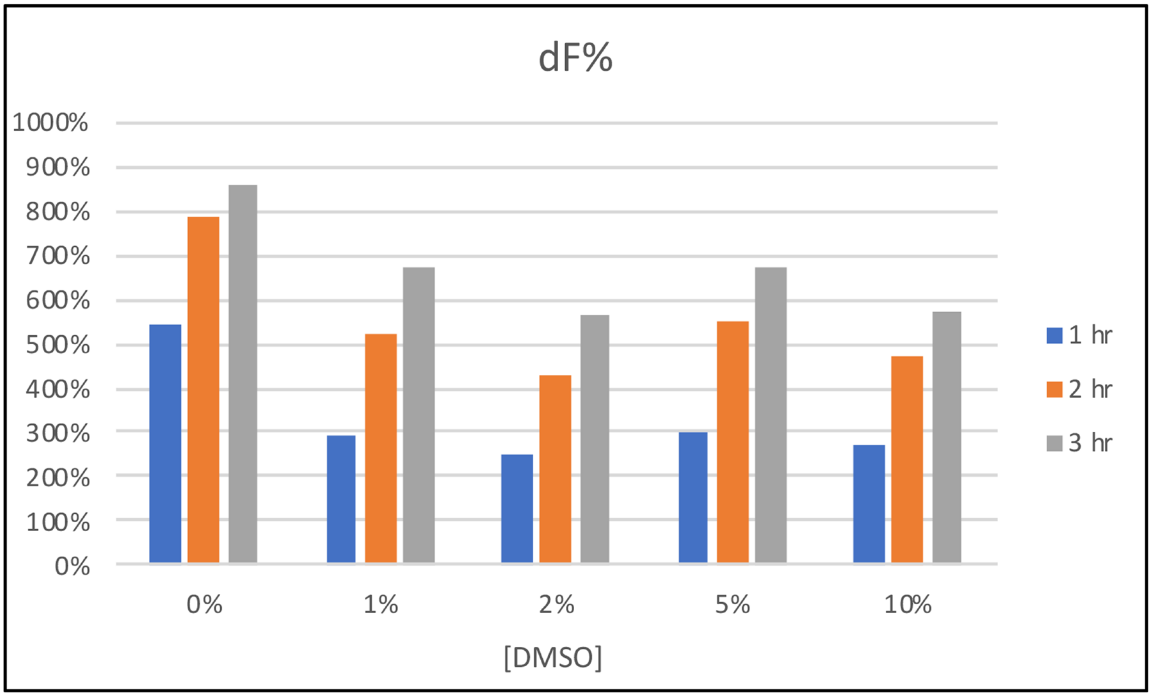

Supplementary Figure 4: Effect of increasing DMSO concentrations on the LMO2-SCL interaction: Normalised FRET ratio dF% resulting from LMO2-SCL PPI in the presence of increasing concentrations of DMSO (0, 1, 2, 5, 10% final concentration). Following addition of the detection antibodies, the plate was read at multiple time points (1, 2, 3 hours post addition) to monitor the effect over time.
